# Association between pre-stroke social participation and current physical resilience across frailty levels among middle-aged and older stroke survivors: A cross-sectional study

**DOI:** 10.1371/journal.pone.0359853

**Published:** 2026-10-06

**Authors:** Yi Zhang, Li Zhang, Jihui Hu, Meng Zhang, Xiapei Peng

**Affiliations:** Department of Neurology, The Central Hospital of Wuhan, Tongji Medical College, Huazhong University of Science and Technology, Wuhan, Hubei, China; University of Valencia: Universitat de Valencia, SPAIN

## Abstract

**Background:**

Physical resilience and frailty are related but distinct constructs that may shape adaptation after stroke. Evidence concerning whether pre-stroke social participation is associated with physical resilience across different frailty levels remains limited.

**Methods:**

This single-center cross-sectional study included 314 adults aged 45 years or older with imaging-confirmed stroke. Participants retrospectively reported seven types of social participation during the six months before stroke. Current frailty and physical resilience were assessed at enrollment using the FRAIL scale and the Physical Resilience Instrument for Older Adults. Participants were classified as non-frail, pre-frail, or frail. Multivariable linear regression with HC3 robust standard errors evaluated the association between social participation and physical resilience. Interaction terms tested whether the association differed by frailty category.

**Results:**

Of 314 participants, 140 (44.6%) were non-frail, 149 (47.5%) were pre-frail, and 25 (8.0%) were frail. Social participation and physical resilience showed a weak positive correlation in the full sample (r = 0.295, 95% CI 0.190 to 0.392; p < 0.001). After adjustment for age, sex, monthly income, hypertension, diabetes, and frailty category, each one-point higher social participation score was associated with a 0.725-point higher physical resilience score (95% CI 0.405 to 1.044; p < 0.001). The social participation-by-frailty interaction was not statistically significant (joint p = 0.314). Results were consistent in analyses restricted to participants aged 60 years or older and 65 years or older.

**Conclusions:**

Retrospectively reported pre-stroke social participation was weakly and positively associated with current physical resilience across frailty levels. The cross-sectional design, retrospective exposure assessment, and limited size of the frail subgroup preclude causal or intervention conclusions.

## 1. Introduction

Stroke remains a major cause of disability and mortality worldwide and contributes substantially to health-care burden in China [1,2]. Recovery after stroke varies markedly, even among patients with apparently similar neurological injury. Physical resilience describes the capacity to maintain or regain functioning after a health stressor and provides a useful framework for understanding this heterogeneity [3,4].

Frailty describes increased vulnerability arising from reduced physiological reserve, whereas physical resilience concerns the capacity to resist or recover from stress. The constructs are related but are not exact opposites [3,5–10]. The FRAIL scale is a brief physical frailty screening instrument addressing fatigue, resistance, ambulation, illnesses, and weight loss [11,12]. Its conventional categories distinguish non-frailty, prefrailty, and frailty, which may differ in both baseline reserve and opportunities for recovery.

Social participation refers to involvement in interpersonal, community, and socially meaningful activities [13]. Frailty can restrict mobility and social engagement, while reduced social participation may coincide with fewer behavioral, emotional, and practical resources [14,15]. Among stroke survivors, social participation is associated with cognitive, psychological, and functional outcomes, but participation is also strongly constrained by disability [16,17]. Assessing current participation after stroke may therefore conflate pre-stroke social resources with consequences of the stroke itself.

Retrospective assessment of activities before stroke offers one way to characterize recent pre-stroke social context, although it remains vulnerable to recall error. Previous work has not established whether the association between pre-stroke social participation and current physical resilience differs between non-frail, pre-frail, and frail stroke survivors. We therefore examined this association in adults across the frailty spectrum and tested frailty category as a potential effect modifier.

## 2. Methods

### 2.1. Study design, setting, and participants

This single-center cross-sectional analysis used enrollment data from an ethics-approved longitudinal project conducted in the Department of Neurology at The Central Hospital of Wuhan, China. Participants were consecutively recruited from February 1 to April 30, 2026. The protocol was approved by the Ethics Committee of The Central Hospital of Wuhan (approval no. WHZXKYL2026−013; January 20, 2026). The study was conducted in accordance with the Declaration of Helsinki, and all participants provided written informed consent before enrollment. STROBE reporting recommendations were followed.

Eligible participants had a stroke confirmed by neuroimaging, were aged 45 years or older, were clinically stable and at least three months after stroke onset, and had a Mini-Mental State Examination score of at least 24 to support completion of self-report measures [18]. Patients were excluded if severe aphasia prevented effective communication, if the Charlson Comorbidity Index was 3 or higher, or if they had a severe pre-existing psychiatric disorder. The comorbidity threshold was prespecified to limit the influence of substantial competing disease burden on self-reported resilience; it was not intended to define a terminal condition. The lower age limit was selected because the parent study included both middle-aged and older stroke survivors; age-restricted sensitivity analyses were conducted because the PRIFOR was developed for older adults.

Frailty and physical resilience were assessed once at enrollment and therefore represent current status. Time since stroke was recorded using three prespecified categories: less than 1 year, 1–5 years, and more than 5 years.

### 2.2. Measures

#### 2.2.1. Physical resilience.

Current physical resilience was assessed with the 16-item Physical Resilience Instrument for Older Adults (PRIFOR) [19–21]. Items are rated on a five-point Likert scale and summed to produce a total score from 16 to 80; higher scores indicate greater perceived physical resilience. The instrument includes positive thinking, coping and lifestyle adjustment, and belief and hope domains. Internal consistency in the present sample was high (Cronbach's alpha = 0.963).

#### 2.2.2. Pre-stroke social participation.

Participants retrospectively reported participation during the six months preceding stroke onset in seven activities: visiting relatives or friends, playing cards or mahjong, helping non-coresident relatives, dancing or exercising in parks, participating in community organizations, undertaking voluntary or charitable work, and providing care to a non-coresident person. The first six items were scored from 0 to 3 according to the study questionnaire, and the caregiving item was scored 0 or 1. Items were summed to yield a social participation score ranging from 0 to 19, with higher scores indicating greater participation. In this sample the observed range was 0–17 and Cronbach's alpha was 0.870. The six-month interval was selected to characterize relatively recent habitual participation; it does not eliminate recall bias.

#### 2.2.3. Frailty.

Current physical frailty was assessed with the five-item FRAIL scale, covering Fatigue, Resistance, Ambulation, Illnesses, and Loss of weight [11,12]. Each component is scored 0 or 1, for a total of 0–5. Participants were classified as non-frail (0), pre-frail (1–2), or frail (3–5).

#### 2.2.4. Covariates.

Covariates available for the prespecified adjusted analysis were age, sex, monthly income, hypertension, and diabetes mellitus. Monthly income was entered as a categorical variable (low, medium, or high). Frailty category was included in the full-cohort model. Pre-stroke physical activity and functional status, stroke severity, acute treatment, rehabilitation dose, and community support services were not comprehensively available and could not be included.

### 2.3. Statistical analysis

Continuous variables are reported as mean (standard deviation), and categorical variables as number (percentage). Characteristics were compared across frailty categories using one-way analysis of variance for continuous variables and chi-square or exact/Monte Carlo tests for categorical variables when sparse cells were present. Pearson correlation coefficients with 95% confidence intervals described unadjusted linear associations. Following Schober et al. [22], correlations between 0.10 and 0.39 were interpreted as weak.

The primary analysis used multivariable linear regression in all 314 participants. Current PRIFOR score was the dependent variable, and pre-stroke social participation score was the primary explanatory variable. The model adjusted for age, sex, monthly income, hypertension, diabetes, and current frailty category. HC3 heteroskedasticity-consistent standard errors were used. Age was mean-centered to aid interpretation of the intercept; variance inflation factors assessed multicollinearity.

Effect modification was examined by adding social participation-by-frailty-category interaction terms and performing a joint two-degree-of-freedom test. Category-specific correlations and parsimonious regression estimates were treated as exploratory because only 25 participants were frail. Sensitivity analyses were restricted to participants aged at least 60 years and at least 65 years and to the combined pre-frail/frail subgroup used in the original analysis. All tests were two-sided, with p < 0.05 considered statistically significant. Analyses were performed in Python 3.8. The author-generated analysis code is supplied without restriction as S1 File; numerical reproduction requires the de-identified dataset available through the controlled-access procedure described in the Data Availability statement.

## 3. Results

### 3.1. Participant characteristics

The analysis included 314 participants: 140 (44.6%) were non-frail, 149 (47.5%) were pre-frail, and 25 (8.0%) were frail. Mean age was 68.4 years (SD 10.0), and 165 participants (52.5%) were male. Mean social participation and physical resilience scores were 5.2 (SD 3.8) and 64.3 (SD 10.9), respectively. Both scores declined across increasing frailty categories (Table 1).

**Table 1:** Participant characteristics according to frailty category (N = 314).

Variable	OverallN = 314	Non-frailn = 140	Pre-frailn = 149	Frailn = 25	P value
Age, years	68.4 (10.0)	66.9 (9.7)	69.3 (10.4)	71.8 (8.1)	0.024
Age group: 45–59	63 (20.1)	33 (23.6)	30 (20.1)	0 (0.0)	0.002
60-69	100 (31.8)	46 (32.9)	46 (30.9)	8 (32.0)	
70-79	115 (36.6)	55 (39.3)	47 (31.5)	13 (52.0)	
>=80	36 (11.5)	6 (4.3)	26 (17.4)	4 (16.0)	
Sex: Male	165 (52.5)	76 (54.3)	80 (53.7)	9 (36.0)	0.224
Female	149 (47.5)	64 (45.7)	69 (46.3)	16 (64.0)	
Education: Primary or below	172 (54.8)	65 (46.4)	93 (62.4)	14 (56.0)	0.005
Secondary	101 (32.2)	56 (40.0)	34 (22.8)	11 (44.0)	
Tertiary or above	41 (13.1)	19 (13.6)	22 (14.8)	0 (0.0)	
Marital status: Married/cohabiting	265 (84.4)	120 (85.7)	122 (81.9)	23 (92.0)	0.276
Divorced/widowed	7 (2.2)	1 (0.7)	6 (4.0)	0 (0.0)	
Single	42 (13.4)	19 (13.6)	21 (14.1)	2 (8.0)	
Monthly income: Low	101 (32.2)	43 (30.7)	46 (30.9)	12 (48.0)	0.031
Medium	159 (50.6)	81 (57.9)	69 (46.3)	9 (36.0)	
High	54 (17.2)	16 (11.4)	34 (22.8)	4 (16.0)	
Time since stroke: < 1 year	174 (55.4)	84 (60.0)	72 (48.3)	18 (72.0)	<0.001
1-5 years	97 (30.9)	47 (33.6)	50 (33.6)	0 (0.0)	
>5 years	43 (13.7)	9 (6.4)	27 (18.1)	7 (28.0)	
Hypertension: No	73 (23.2)	41 (29.3)	29 (19.5)	3 (12.0)	0.054
Yes	241 (76.8)	99 (70.7)	120 (80.5)	22 (88.0)	
Diabetes mellitus: No	214 (68.2)	105 (75.0)	98 (65.8)	11 (44.0)	0.006
Yes	100 (31.8)	35 (25.0)	51 (34.2)	14 (56.0)	
Coronary heart disease: No	261 (83.1)	120 (85.7)	116 (77.9)	25 (100.0)	0.013
Yes	53 (16.9)	20 (14.3)	33 (22.1)	0 (0.0)	
Pre-stroke social participation score	5.2 (3.8)	6.2 (3.7)	4.5 (3.4)	3.0 (4.7)	<0.001
Current physical resilience score	64.3 (10.9)	67.1 (8.5)	62.8 (12.2)	57.6 (9.6)	<0.001

Values are mean (SD) or n (%). P values compare the three frailty categories. Monte Carlo conditional tests based on 100,000 tables were used when any expected cell count was below 5. Time since stroke was analyzed as a categorical variable, not as continuous years.

### 3.2. Social participation and physical resilience

In the full sample, pre-stroke social participation and current physical resilience were weakly positively correlated (r = 0.295, 95% CI 0.190 to 0.392; p < 0.001). The corresponding correlations were r = 0.224 (p = 0.008) in non-frail participants, r = 0.267 (p = 0.001) in pre-frail participants, and r = 0.245 (p = 0.237) in frail participants. The frail estimate had a wide 95% confidence interval (−0.166 to 0.584). Fig 1 displays the category-specific data and fitted lines.

**Fig 1:**
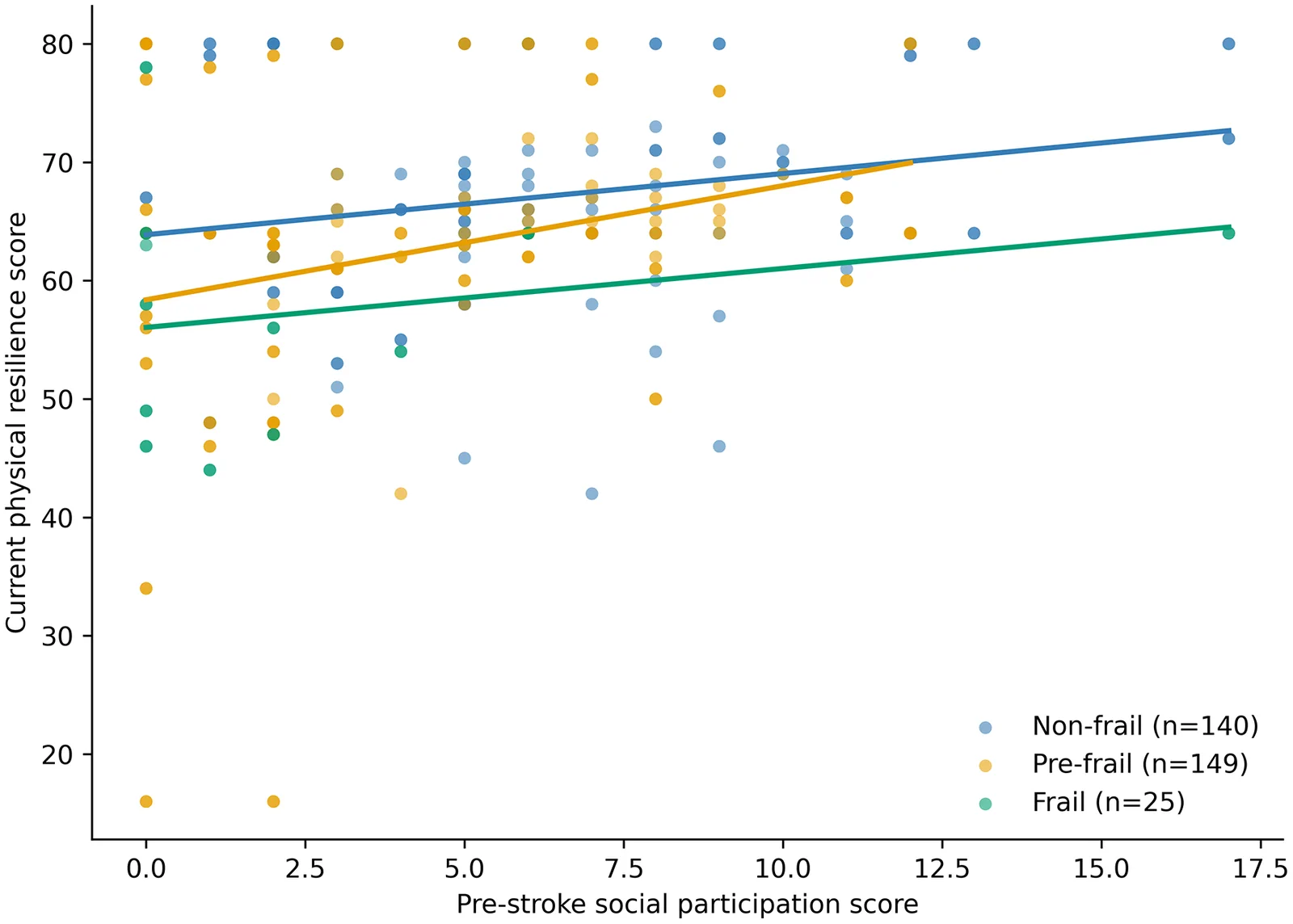
Association between pre-stroke social participation and current physical resilience according to frailty category. Points represent individual participants; lines are unadjusted category-specific linear fits.

### 3.3. Adjusted association

In the primary adjusted model, each one-point higher social participation score was associated with a 0.725-point higher current physical resilience score (95% CI 0.405 to 1.044; p < 0.001; Table 2). The model explained 21.2% of outcome variance (adjusted R2 = 0.188). The maximum variance inflation factor was 1.36.

**Table 2:** Multivariable linear regression of current physical resilience in the full cohort.

Variable	B	HC3 SE	95% CI	P value
Constant	65.739	2.178	61.453 to 70.025	<0.001
Age (centered)	−0.101	0.056	−0.211 to 0.008	0.069
Female sex	−1.468	1.243	−3.913 to 0.978	0.238
Medium income	0.803	1.337	−1.828 to 3.434	0.548
High income	3.078	1.865	−0.592 to 6.749	0.100
Hypertension	−3.404	1.698	−6.745 to −0.063	0.046
Diabetes mellitus	−4.182	1.240	−6.623 to −1.741	<0.001
Pre-frail	−2.405	1.104	−4.578 to −0.232	0.030
Frail	−4.542	2.239	−8.948 to −0.137	0.043
Social participation score	0.725	0.162	0.405 to 1.044	<0.001

Reference categories: male sex, low income, no hypertension, no diabetes, and non-frail. B values are unstandardized coefficients. R2 = 0.212; adjusted R2 = 0.188.

### 3.4. Effect modification and sensitivity analyses

The interaction terms were not statistically significant: social participation x pre-frail B = 0.494 (95% CI −0.211 to 1.198; p = 0.169) and social participation x frail B = 0.358 (95% CI −0.303 to 1.020; p = 0.287). The joint interaction test was also not significant (p = 0.314), providing no statistical evidence that the association differed by frailty category. This absence of evidence should be interpreted cautiously because the frail subgroup was small.

In age-restricted analyses, the adjusted coefficients for social participation were 0.894 (95% CI 0.452 to 1.337; p < 0.001) among 251 participants aged at least 60 years and 0.902 (95% CI 0.396 to 1.408; p < 0.001) among 213 participants aged at least 65 years. In the combined pre-frail/frail subgroup (n = 174), the adjusted coefficient was 0.990 (95% CI 0.501 to 1.478; p < 0.001). Full sensitivity results are provided in S1 Appendix.

## 4. Discussion

In this cross-sectional sample of middle-aged and older stroke survivors, retrospectively reported pre-stroke social participation was weakly and positively associated with current physical resilience. The adjusted association remained after accounting for measured demographic factors, hypertension, diabetes, and current frailty category. We found no statistical evidence that the association varied across non-frail, pre-frail, and frail categories, although the estimate in the small frail subgroup was imprecise.

The findings should be interpreted in light of the conceptual distinction between frailty and resilience. Frailty reflects vulnerability and reduced reserve, whereas resilience concerns adaptation to a stressor [3,4,8]. Their coexistence is therefore plausible. The lower adjusted PRIFOR scores in pre-frail and frail participants support relatedness between the constructs but do not establish that they are interchangeable. Including the full frailty spectrum avoids defining the analytic cohort by a current characteristic that is closely related to the outcome.

The correlation magnitude was weak rather than clinically meaningful [22]. Social participation is only one component of a broader social and functional context, and the adjusted model explained a limited proportion of variation in resilience. Furthermore, a minimal clinically important difference for the PRIFOR has not been established in stroke survivors. The coefficient should therefore be viewed as an association estimate, not evidence of a clinically important intervention effect.

Broader healthy-aging frameworks situate adaptive capacity within intrinsic capacity and environmental context [23,24]. Several mechanisms may explain the observed association, but they were not tested here. Social activities may accompany greater pre-stroke mobility, cognitive engagement, health behavior, and access to emotional or practical support [13,16,17,25–29]. Conversely, people with better pre-stroke function may be more able to participate socially. This confounding by prior function is especially important in frail populations, in whom mobility limitations and social isolation frequently coexist.

Clinically, a brief social history may complement assessment of medical and functional factors and identify potential barriers to community reintegration. The findings do not demonstrate that post-stroke social prescribing improves physical resilience. Social prescribing and peer or community programs remain plausible approaches, but their accessibility, safety, timing, and effectiveness for people with frailty and mobility limitation require prospective evaluation [30–34].

This study has several limitations. First, its cross-sectional design cannot establish temporal or causal effects, a recovery trajectory, or directionality. Second, pre-stroke participation was recalled after stroke and may be affected by recall error, mood, cognition, or current health. Third, frailty and resilience were measured concurrently, and the constructs have conceptual and item-level overlap. Fourth, pre-stroke physical activity and functioning, stroke severity, treatment and treatment timing, rehabilitation intensity and duration, depression, and community support services were not comprehensively measured; residual confounding is therefore likely. Fifth, the single-center hospital sample and exclusions related to cognition, aphasia, severe psychiatric illness, and CCI scores of 3 or higher restrict generalizability, particularly to community-dwelling survivors, patients with greater impairment, and those with substantial multimorbidity. Sixth, the PRIFOR was developed for older adults, although age-restricted analyses were consistent. Finally, only 25 participants were frail, limiting precision for interaction and stratum-specific estimates.

## 5. Conclusions

Retrospectively reported pre-stroke social participation was weakly and positively associated with current physical resilience among middle-aged and older stroke survivors after adjustment for measured covariates and frailty category. There was no statistical evidence that the association differed across frailty levels, but the frail subgroup estimate was imprecise. Prospective, multicenter studies incorporating pre-stroke function, stroke severity, treatment, rehabilitation exposure, and community support are needed before intervention or prognostic implications can be established.

## Supporting information

S1 Fig Normal Q-Q plot of residuals from the primary multivariable linear regression model. (TIF)

S1 Appendix Supplementary correlation, interaction, subgroup, and age-restricted sensitivity analyses. (DOCX)

S1 File Author-generated Python code for data-quality and scale-scoring checks, descriptive statistics, regression models, sensitivity analyses, model diagnostics, and figures. Execution requires the de-identified dataset available through the controlled-access procedure described in the Data Availability statement.
(PY)
