# Supplementary material for: Association between pre-stroke social participation and current physical resilience across frailty levels among middle-aged and older stroke survivors: A cross-sectional study
Source: PLoS One. 2026 Oct 6;21(10):e0359853. doi: 10.1371/journal.pone.0359853 (PMC13641336; doi:10.1371/journal.pone.0359853)
Supplement: Supplementary file 1 [file pone.0359853.s002.docx]

S1 Appendix. Supplementary analyses

Association between pre-stroke social participation and current physical resilience across frailty levels among middle-aged and older stroke survivors: A cross-sectional study

# S1 Table. Pearson correlations by frailty category

| Analysis group | n | r | 95% CI | P value |
| --- | --- | --- | --- | --- |
| Overall | 314 | 0.295 | 0.190 to 0.392 | <0.001 |
| Non-frail | 140 | 0.224 | 0.061 to 0.376 | 0.008 |
| Pre-frail | 149 | 0.267 | 0.110 to 0.410 | 0.001 |
| Frail | 25 | 0.245 | -0.166 to 0.584 | 0.237 |
| Pre-frail/frail combined | 174 | 0.274 | 0.131 to 0.406 | <0.001 |

The frail-stratum estimate is imprecise because only 25 participants were classified as frail.

# S2 Table. Interaction between social participation and frailty category

| Term | B | HC3 SE | 95% CI | P value |
| --- | --- | --- | --- | --- |
| Social participation score | 0.472 | 0.186 | 0.106 to 0.838 | 0.012 |
| SPS × pre-frail | 0.494 | 0.358 | -0.211 to 1.198 | 0.169 |
| SPS × frail | 0.358 | 0.336 | -0.303 to 1.020 | 0.287 |

Joint test of the two interaction terms: chi-square=2.318, df=2, p=0.314. Reference category: non-frail. In the interaction model, the coefficient for social participation represents its conditional association in the non-frail reference category and therefore differs from the coefficient in the primary model without interaction terms.

# S3 Table. Sensitivity analyses

| Analysis | n | B | 95% CI | P value |
| --- | --- | --- | --- | --- |
| Age ≥ 60 | 251 | 0.894 | 0.452 to 1.337 | <0.001 |
| Age ≥ 65 | 213 | 0.902 | 0.396 to 1.408 | <0.001 |
| Pre-frail/frail combined | 174 | 0.990 | 0.501 to 1.478 | <0.001 |

All models used HC3 robust standard errors. Age-restricted models adjusted for age, sex, monthly income, hypertension, diabetes, and frailty category. The combined pre-frail/frail model adjusted for age, sex, monthly income, hypertension, and diabetes.
