# Supplementary material for: Association between pre-stroke social participation and current physical resilience across frailty levels among middle-aged and older stroke survivors: A cross-sectional study
Source: PLoS One. 2026 Oct 6;21(10):e0359853. doi: 10.1371/journal.pone.0359853 (PMC13641336; doi:10.1371/journal.pone.0359853)
Supplement: Supplementary file 2 [file pone.0359853.s005.docx]

**Response to the Editor and Reviewers**

**Manuscript ID:** PONE-D-26-23300R1

**Revised title:** Association between pre-stroke social participation and current physical resilience across frailty levels among middle-aged and older stroke survivors: A cross-sectional study

Dear Dr. Ingles,

Thank you for the careful editorial assessment and constructive reviews. We have substantially revised the manuscript and reanalyzed data from all 314 included participants. The principal changes are: analysis of the full frailty spectrum; explicit frailty-category interaction testing; age-restricted and pre-frail/frail sensitivity analyses; correction of time since stroke as a categorical variable; full description of all seven SPS items and their scoring; clarification of the CCI exclusion threshold; and expanded discussion of design, confounding, generalizability, and clinical implications. We have also supplied analysis code and separate supporting-information files. Our point-by-point responses follow.

**Note:** Page and line numbers below refer to the final reformatted marked manuscript (16 pages, 303 numbered lines). The clean and marked manuscripts have identical pagination. Reviewer comments are reproduced in black italics, our responses and locations in blue, and revised manuscript text in dark red.

# Response to the Journal Requirements

## E1.

**Comment:** *Please ensure that the manuscript meets PLOS ONE style requirements, including file naming.*

**Response:** We reformatted the submission as separate journal-ready files: an unmarked manuscript, a marked manuscript, a Response to Reviewers, separate figure files, S1 Appendix, and S1 File containing the analysis code. Supporting-information legends are retained in the manuscript.

**Location in the revised manuscript:** Throughout the submission package; Supporting Information Legends appear after the references on p. 16, lines 296–303.

**Revised text:** ‘S1 Fig. Normal Q-Q plot of residuals from the primary multivariable linear regression model. S1 Appendix. Supplementary correlation, interaction, subgroup, and age-restricted sensitivity analyses. S1 File. Author-generated Python code for data-quality and scale-scoring checks, descriptive statistics, regression models, sensitivity analyses, model diagnostics, and figures.’

## E2.

**Comment:** *PLOS ONE requires author-generated code underpinning the findings to be made available without restrictions upon publication.*

**Response:** We agree. We have prepared the author-generated Python analysis code as S1 File and will make it available without restrictions. The code contains no participant identifiers or participant-level data. When used with the de-identified dataset obtained through the controlled-access procedure described in the Data Availability statement, the code reproduces data-quality and scale-scoring checks, descriptive statistics, the primary and interaction models, sensitivity analyses, model diagnostics, and figures. Because the participants' written informed consent did not authorize unrestricted public disclosure of individual-level clinical data, the dataset itself is not included with the code.

**Location in the revised manuscript:** Data Availability statement, p. 1, lines 11–18; Statistical analysis, p. 6, lines 130–132; Supporting Information Legends, p. 16, lines 296–303.

**Revised text:** ‘The author-generated Python analysis code is provided without restriction as S1 File. Execution of the code requires the de-identified dataset obtained through this controlled-access procedure.’

## E3.

**Comment:** *The ethical approval number in the manuscript/submission metadata does not match the ethical approval document.*

**Response:** We verified the ethics document and standardized the correct approval number and date throughout the manuscript and submission materials: WHZXKYL2026-013, January 20, 2026. We will enter the same information in Editorial Manager.

**Location in the revised manuscript:** Methods, p. 4, lines 69–73.

**Revised text:** ‘The protocol was approved by the Ethics Committee of The Central Hospital of Wuhan (WHZXKYL2026-013; January 20, 2026). All participants provided written informed consent before enrollment.’

## E4.

**Comment:** *Supplementary figures should be removed from the manuscript and uploaded as Supporting Information, with legends listed after the references.*

**Response:** We removed the supplementary diagnostic figure from the manuscript body and prepared it as a separate S1 Fig file. Its legend, together with the S1 Appendix and S1 File legends, is listed in the manuscript.

**Location in the revised manuscript:** Supporting Information Legends, p. 16, lines 296–303; separate S1 Fig file.

**Revised text:** ‘S1 Fig. Normal Q-Q plot of residuals from the primary multivariable linear regression model.’

## E5.

**Comment:** *Please include the full ethics statement in Methods, including the approving committee and whether written or verbal consent was obtained.*

**Response:** We added the study design, approving institution, approval identifier and date, compliance statement, and written-consent information directly to Methods section 2.1. The parent project was longitudinal, whereas the present report is explicitly identified as a cross-sectional analysis of enrollment data.

**Location in the revised manuscript:** Methods, p. 4, lines 69–73.

**Revised text:** ‘The protocol was approved by the Ethics Committee of The Central Hospital of Wuhan (approval no. WHZXKYL2026-013; January 20, 2026). The study was conducted in accordance with the Declaration of Helsinki, and all participants provided written informed consent before enrollment.’

## E6.

**Comment:** *Please explain any ethical/legal restrictions on sharing de-identified data, identify who imposed them, and provide contact information; otherwise deposit the minimal dataset publicly.*

**Response:** The written consent did not authorize unrestricted public release of individual-level clinical data, and the ethics-approved terms require institutional review of external transfer. We therefore cannot ethically place the participant-level dataset in an unrestricted repository. We now identify the institutional decision-maker, give its postal address and telephone number, and explain the criteria for access. De-identified data necessary to reproduce the findings may be requested from the Ethics Committee and corresponding author. We will reproduce this wording in the submission system. The author-generated analysis code is unrestricted in S1 File, contains no participant-level data, and requires the controlled-access dataset for execution.

**Location in the revised manuscript:** Data Availability statement, p. 1, lines 11–18.

**Revised text:** ‘Individual-level data cannot be deposited publicly because the written consent did not authorize unrestricted public sharing and the ethics-approved terms require institutional review of external data transfer. De-identified data necessary to reproduce the findings may be requested from the Ethics Committee of The Central Hospital of Wuhan (26 Shengli Street, Jiang'an District, Wuhan, Hubei 430014, China; telephone: +86-27-82223802) and the corresponding author. Requests will be assessed for scientific purpose, participant privacy, and compliance with the approved consent.’

## E7.

**Comment:** *Reviewer-recommended publications should be evaluated for relevance; citation is not automatically required.*

**Response:** We evaluated the recommended Schober et al. article and found it directly relevant to interpretation of correlation magnitude. We cited the peer-reviewed publication and used its thresholds. We did not use or cite the unofficial Sci-Hub link included in the review.

**Location in the revised manuscript:** Statistical analysis, p. 6, lines 116–119; Discussion, p. 11, lines 184–188; Reference 22, p. 15, lines 267–268.

**Revised text:** ‘Following Schober et al. [22], correlations between 0.10 and 0.39 were interpreted as weak.’

# Response to the Additional Editor Comments

## AE1.

**Comment:** *Please remove the duplicated statement regarding written informed consent in Section 2.1.*

**Response:** We removed the duplicate. Written consent is now stated once, within the complete ethics statement in Methods section 2.1.

**Location in the revised manuscript:** Methods, p. 4, lines 69–73.

**Revised text:** ‘All participants provided written informed consent before enrollment.’

## AE2.

**Comment:** *Please ensure consistency of the ethics approval information throughout the submission.*

**Response:** We verified and standardized the correct committee name, approval number, and date in all files and will use the same metadata at resubmission.

**Location in the revised manuscript:** Methods, p. 4, lines 69–73.

**Revised text:** ‘Ethics Committee of The Central Hospital of Wuhan (approval no. WHZXKYL2026-013; January 20, 2026).’

## AE3.

**Comment:** *Please ensure consistency in the Data Availability Statement regarding restrictions and reasonable-access procedures.*

**Response:** We replaced inconsistent wording with one detailed statement explaining the consent/ethics restriction, the institutional review procedure, the contacts, and the availability of reproducible code.

**Location in the revised manuscript:** Data Availability statement, p. 1, lines 11–18.

**Revised text:** ‘Requests will be assessed for scientific purpose, participant privacy, and compliance with the approved consent. The author-generated Python analysis code is provided without restriction as S1 File.’

## AE4.

**Comment:** *Please review the manuscript globally for consistent use of ‘frail,’ ‘pre-frail/frail,’ ‘physical resilience,’ ‘association,’ and ‘prediction.’*

**Response:** We reviewed the manuscript globally. We use non-frail, pre-frail, and frail for the three FRAIL categories; pre-frail/frail only for the combined sensitivity subgroup; current physical resilience for the outcome; and association rather than prediction or causal terminology.

**Location in the revised manuscript:** Title and throughout; group definitions on p. 5, lines 103–106; analysis on p. 6, lines 120–132.

**Revised text:** ‘Participants were classified as non-frail (0), pre-frail (1–2), or frail (3–5).’

## AE5.

**Comment:** *Please revise the title if the analysis includes both pre-frail and frail participants.*

**Response:** We revised the title to state that the full frailty spectrum was studied, consistent with the primary analysis of all 314 participants.

**Location in the revised manuscript:** Title, p. 1, lines 1–2.

**Revised text:** ‘Association between pre-stroke social participation and current physical resilience across frailty levels among middle-aged and older stroke survivors: A cross-sectional study.’

# Response to Reviewer 1

## R1.1.

**Comment:** *Frailty is mentioned only briefly. Please explain frailty more clearly, identify the model used, and help readers understand the significance of its factors for the outcome.*

**Response:** We expanded the conceptual framework. We distinguish frailty (vulnerability/reduced reserve) from physical resilience (capacity to resist or recover), explain that they are related but not exact opposites, and describe the FRAIL scale as a brief physical frailty screening instrument. The scale operationalizes five physical/deficit domains rather than the Fried phenotype or Rockwood accumulation index; we therefore avoid labeling it as either model.

**Location in the revised manuscript:** Introduction, p. 3, lines 48–53; Frailty measure, p. 5, lines 103–106; Discussion, p. 11, lines 178–183.

**Revised text:** ‘Frailty describes increased vulnerability arising from reduced physiological reserve, whereas physical resilience concerns the capacity to resist or recover from stress. The constructs are related but are not exact opposites.’

## R1.2.

**Comment:** *Strengthen the problem formulation and justify why pre-stroke social participation may matter particularly in relation to frailty, functional ability, isolation, and resilience.*

**Response:** We strengthened the rationale while avoiding a causal claim. The revised Introduction explains that frailty may restrict mobility and participation, whereas lower participation may coincide with fewer behavioral, emotional, and practical resources. It also notes that post-stroke participation can be a consequence of disability, motivating assessment of the pre-stroke period. The Discussion addresses reverse causation and prior function explicitly.

**Location in the revised manuscript:** Introduction, p. 3, lines 54–64; Discussion, pp. 11–12, lines 189–200.

**Revised text:** ‘Assessing current participation after stroke may therefore conflate pre-stroke social resources with consequences of the stroke itself. Retrospective assessment of activities before stroke offers one way to characterize recent pre-stroke social context, although it remains vulnerable to recall error.’

## R1.3.

**Comment:** *The aim refers to an ‘independent association.’ What does this mean, and how can independence be determined?*

**Response:** We removed ambiguous ‘independent’ language from the title, aim, and conclusions. We now describe an adjusted association and specify every covariate. This means conditional statistical adjustment for measured variables; it does not establish causal independence from unmeasured factors.

**Location in the revised manuscript:** Abstract, pp. 1–2, lines 20–40; Methods, p. 6, lines 120–124; Conclusions, pp. 12–13, lines 213–219.

**Revised text:** ‘After adjustment for age, sex, monthly income, hypertension, diabetes, and frailty category, each one-point higher social participation score was associated with a 0.725-point higher physical resilience score (95% CI 0.405 to 1.044; p<0.001).’

## R1.4.

**Comment:** *How do you justify including participants aged 45 years and older while framing the study around frailty and using a geriatric measure?*

**Response:** The lower age limit came from the parent study, which enrolled middle-aged and older stroke survivors. We now state this transparently and avoid presenting the cohort as exclusively geriatric. Because PRIFOR was developed for older adults, we added prespecified sensitivity analyses among participants aged at least 60 and 65 years. The association remained: age ≥60, n=251, B=0.894 (95% CI 0.452–1.337; p<0.001); age ≥65, n=213, B=0.902 (95% CI 0.396–1.408; p<0.001). This consistency supports robustness but does not itself validate PRIFOR in younger participants.

**Location in the revised manuscript:** Methods, p. 4, lines 74–85 and p. 6, lines 125–132; Results, p. 10, lines 166–170; Discussion, p. 12, lines 210–212.

**Revised text:** ‘The lower age limit was selected because the parent study included both middle-aged and older stroke survivors; age-restricted sensitivity analyses were conducted because the PRIFOR was developed for older adults.’

## R1.5.

**Comment:** *Why exclude participants with CCI ≥3? This threshold may exclude the most frail individuals.*

**Response:** We agree that CCI ≥3 is not synonymous with terminal illness and that this criterion may reduce representation of multimorbidity and frailty. We restored the exact prespecified threshold, explained its original purpose—to limit substantial competing disease burden in self-reported resilience—and explicitly state that it was not a terminal-condition definition. Because this design choice cannot be reversed in the available cohort, we acknowledge selection and restricted generalizability rather than claiming it was optimal.

**Location in the revised manuscript:** Methods, p. 4, lines 74–82; Limitations, p. 12, lines 201–212.

**Revised text:** ‘Patients were excluded ... if the Charlson Comorbidity Index was 3 or higher... The comorbidity threshold was prespecified to limit the influence of substantial competing disease burden on self-reported resilience; it was not intended to define a terminal condition.’

## R1.6.

**Comment:** *How do the ≥3-month post-stroke criterion and six-month pre-stroke recall window minimize recall bias?*

**Response:** We removed the unsupported claim that these intervals minimize recall bias. The ≥3-month criterion defines the post-acute eligible population; the six-month window was intended to characterize relatively recent habitual participation. Neither eliminates retrospective error. We now identify recall bias as a central limitation. We also corrected disease duration: the source data record time since stroke only as <1 year (n=174), 1–5 years (n=97), or >5 years (n=43), so we report categories and do not calculate a spurious mean duration.

**Location in the revised manuscript:** Methods, pp. 4–5, lines 74–85 and 93–102; Table 1, pp. 7–8, with note at lines 140–142; Limitations, p. 12, lines 201–207.

**Revised text:** ‘Time since stroke was recorded using three prespecified categories: less than 1 year, 1–5 years, and more than 5 years.’ and ‘The six-month interval was selected to characterize relatively recent habitual participation; it does not eliminate recall bias.’

## R1.7.

**Comment:** *Provide a reference for the validated FRAIL instrument and describe its model and domains.*

**Response:** We added the original FRAIL publication and international guideline references, spelled out all five domains, and specified scoring and thresholds. We describe it accurately as a physical frailty screening instrument rather than assigning it to a different frailty model.

**Location in the revised manuscript:** Introduction, p. 3, lines 48–53; Methods, p. 5, lines 103–106; References 11–12, p. 14, lines 241–244.

**Revised text:** ‘Current physical frailty was assessed with the five-item FRAIL scale, covering Fatigue, Resistance, Ambulation, Illnesses, and Loss of weight [11,12]. Each component is scored 0 or 1, for a total of 0–5.’

## R1.8.

**Comment:** *Please consider frailty and functional ability as covariates.*

**Response:** We revised the primary analysis to include current frailty category as a covariate in all 314 participants. The adjusted social-participation coefficient was B=0.725 (95% CI 0.405–1.044; p<0.001). Pre-stroke physical activity and functional status were not comprehensively measured and therefore cannot be added retrospectively; we now state this in Methods and treat residual confounding as a major limitation.

**Location in the revised manuscript:** Covariates and analysis, pp. 5–6, lines 107–132; Table 2, pp. 9–10; Limitations, p. 12, lines 201–212.

**Revised text:** ‘Frailty category was included in the full-cohort model. Pre-stroke physical activity and functional status, stroke severity, acute treatment, rehabilitation dose, and community support services were not comprehensively available and could not be included.’

## R1.9.

**Comment:** *The reported correlation should be considered weak; discuss it using Schober et al.*

**Response:** We agree. Reanalysis of all 314 records yielded r=0.295 (95% CI 0.190–0.392; p<0.001), which is weak by Schober et al. We cite that paper, consistently use ‘weak,’ report confidence intervals, and caution that statistical significance is not clinical importance. The model explained 21.2% of outcome variance, and an established PRIFOR minimal clinically important difference in stroke survivors is unavailable.

**Location in the revised manuscript:** Methods, p. 6, lines 116–119; Results, p. 9, lines 143–156; Discussion, p. 11, lines 184–188; Reference 22, p. 15, lines 267–268.

**Revised text:** ‘The correlation magnitude was weak rather than clinically meaningful... The coefficient should therefore be viewed as an association estimate, not evidence of a clinically important intervention effect.’

## R1.10.

**Comment:** *Provide a balanced, critical interpretation for frail patients, including social isolation and reduced functional capacity before stroke.*

**Response:** We expanded the Discussion to explain that low mobility and social isolation may precede stroke and confound the observed association. We also report the imprecision of the frail-only estimate: r=0.245, p=0.237, 95% CI −0.166 to 0.584, with only 25 frail participants. Thus, we do not infer absence of association or equivalence across categories from the nonsignificant frail stratum.

**Location in the revised manuscript:** Results, p. 9, lines 143–148; Discussion, pp. 11–12, lines 171–195 and 201–212.

**Revised text:** ‘Conversely, people with better pre-stroke function may be more able to participate socially. This confounding by prior function is especially important in frail populations, in whom mobility limitations and social isolation frequently coexist.’

## R1.11.

**Comment:** *Develop the clinical implications: what can society, health systems, and related sectors do to support participation and resilience?*

**Response:** We added cautious, actionable implications. A brief social history may identify barriers to community reintegration, and community, peer, and social-prescribing approaches merit prospective evaluation. Because the design is cross-sectional, we explicitly do not claim that increasing participation will improve resilience, and we emphasize accessibility and safety for people with frailty or mobility limitations.

**Location in the revised manuscript:** Discussion, p. 12, lines 196–200.

**Revised text:** ‘Clinically, a brief social history may complement assessment of medical and functional factors and identify potential barriers to community reintegration... Social prescribing and peer or community programs remain plausible approaches, but their accessibility, safety, timing, and effectiveness for people with frailty and mobility limitation require prospective evaluation.’

# Response to Reviewer 2

## R2.1.

**Comment:** *This single-centre, hospital-based study cannot be generalized to community-dwelling survivors or other populations.*

**Response:** We agree and now identify the sampling frame as a principal limitation. We narrowed the conclusions and call for multicenter prospective research.

**Location in the revised manuscript:** Abstract, p. 2, lines 37–40; Methods, pp. 3–4, lines 66–73; Limitations, p. 12, lines 207–212; Conclusions, pp. 12–13, lines 213–219.

**Revised text:** ‘The single-center hospital sample and exclusions related to cognition, aphasia, severe psychiatric illness, and CCI scores of 3 or higher restrict generalizability, particularly to community-dwelling survivors, patients with greater impairment, and those with substantial multimorbidity.’

## R2.2.

**Comment:** *Discuss in greater detail the limitations of the cross-sectional design and retrospective pre-stroke assessment.*

**Response:** We expanded these limitations: no temporal or causal inference, no recovery trajectory, possible reverse directionality, and recall error affected by mood, cognition, or current health. We consistently describe retrospectively reported pre-stroke participation and current resilience.

**Location in the revised manuscript:** Abstract, p. 2, lines 37–40; Discussion, p. 12, lines 201–207; Conclusions, pp. 12–13, lines 213–219.

**Revised text:** ‘Its cross-sectional design cannot establish temporal or causal effects, a recovery trajectory, or directionality. Second, pre-stroke participation was recalled after stroke and may be affected by recall error, mood, cognition, or current health.’

## R2.3.

**Comment:** *At what post-stroke time point were frailty and resilience assessed?*

**Response:** Both measures were administered once at study enrollment, at least three months after stroke. The available source data do not record an exact continuous interval; they record <1 year, 1–5 years, or >5 years. We now report these categories and counts instead of interpreting category codes as years.

**Location in the revised manuscript:** Methods, p. 4, lines 74–85; Table 1, pp. 7–8, with note at p. 8, lines 140–142.

**Revised text:** ‘Frailty and physical resilience were assessed once at enrollment and therefore represent current status. Time since stroke was recorded using three prespecified categories: less than 1 year, 1–5 years, and more than 5 years.’

## R2.4.

**Comment:** *Pre-frail and frail participants were incorrectly called the ‘frail group’; use ‘pre-frail/frail group.’*

**Response:** We corrected terminology throughout. The primary analysis now retains three separate categories. ‘Pre-frail/frail’ is used only for the combined sensitivity analysis, not as a synonym for frail.

**Location in the revised manuscript:** Frailty definitions, p. 5, lines 103–106; Results, p. 10, lines 160–170; throughout.

**Revised text:** ‘Participants were classified as non-frail (0), pre-frail (1–2), or frail (3–5).’

## R2.5.

**Comment:** *Justify analyzing pre-frail and frail patients together; resilience may be lower in the frail group.*

**Response:** We accepted the concern and changed the analytical strategy. The primary model includes all 314 participants and adjusts for separate pre-frail and frail indicators. Table 1 displays lower mean resilience across categories (non-frail 67.1, pre-frail 62.8, frail 57.6). The former combined group is retained only as a sensitivity analysis (n=174; SPS B=0.990, 95% CI 0.501–1.478; p<0.001) for continuity with the original submission.

**Location in the revised manuscript:** Methods, p. 6, lines 120–132; Table 1, pp. 7–8; Results, pp. 9–10, lines 152–170.

**Revised text:** ‘The primary analysis used multivariable linear regression in all 314 participants... Sensitivity analyses were restricted... to the combined pre-frail/frail subgroup used in the original analysis.’

## R2.6.

**Comment:** *Study the full frailty spectrum, stratify by non-frail, pre-frail, and frail, and determine whether the association is similar in non-frail survivors.*

**Response:** We implemented this recommendation. Category-specific correlations were non-frail r=0.224 (p=0.008), pre-frail r=0.267 (p=0.001), and frail r=0.245 (p=0.237; 95% CI −0.166 to 0.584). Formal interaction terms were nonsignificant (SPS×pre-frail p=0.169; SPS×frail p=0.287; joint p=0.314). These data provide no statistical evidence of effect modification, but the frail subgroup (n=25) is too small for a precise conclusion. We also corrected the conceptual statement: resilience and frailty are related, not simple opposites.

**Location in the revised manuscript:** Methods, p. 6, lines 120–132; Results, p. 9, lines 143–156 and p. 10, lines 160–170; Discussion, p. 11, lines 171–188.

**Revised text:** ‘We found no statistical evidence that the association varied across non-frail, pre-frail, and frail categories, although the estimate in the small frail subgroup was imprecise.’

## R2.7.

**Comment:** *Pre-stroke physical activity/functioning is an important confounder and should be discussed.*

**Response:** We agree. These variables were not comprehensively collected, so adjustment would create unsupported or incomplete measures. We added this fact to Methods and explain possible reverse causation and residual confounding in Discussion.

**Location in the revised manuscript:** Covariates, p. 5, lines 107–112; Discussion, pp. 11–12, lines 189–195 and 201–212.

**Revised text:** ‘Conversely, people with better pre-stroke function may be more able to participate socially.’

## R2.8.

**Comment:** *Treatment, treatment timing, rehabilitation intensity/duration, and community support services may confound recovery and resilience.*

**Response:** We agree. These factors were not comprehensively available in the dataset, so they cannot be reconstructed honestly. We list them explicitly as unmeasured variables and recommend that future prospective studies collect them.

**Location in the revised manuscript:** Covariates, p. 5, lines 107–112; Limitations, p. 12, lines 201–212; Conclusions, pp. 12–13, lines 213–219.

**Revised text:** ‘Pre-stroke physical activity and functioning, stroke severity, treatment and treatment timing, rehabilitation intensity and duration, depression, and community support services were not comprehensively measured; residual confounding is therefore likely.’

## R2.9.

**Comment:** *The informed-consent sentence is duplicated in Methods.*

**Response:** We removed the duplicate and retained one complete, consistent statement specifying written informed consent.

**Location in the revised manuscript:** Methods, p. 4, lines 69–73.

**Revised text:** ‘All participants provided written informed consent before enrollment.’

# Additional Author Clarification

## AC1.

**Comment:** *Clarify the seven SPS items and their scoring so that the exposure is reproducible.*

**Response:** We verified the source data and now list all seven activities. The first six variables are coded 0–3, caregiving is binary (0/1), and the theoretical total is 0–19; the observed sample range is 0–17. The dataset does not retain the verbal frequency anchors for 0–3, so we report the verified numeric scoring and do not invent labels.

**Location in the revised manuscript:** Pre-stroke social participation measure, p. 5, lines 93–102.

**Revised text:** ‘Participants retrospectively reported... seven activities: visiting relatives or friends, playing cards or mahjong, helping non-coresident relatives, dancing or exercising in parks, participating in community organizations, undertaking voluntary or charitable work, and providing care to a non-coresident person. The first six items were scored from 0 to 3 according to the study questionnaire, and the caregiving item was scored 0 or 1.’

## AC2.

**Comment:** *Ensure that the multicollinearity diagnostic is consistent with the final design matrix and shared analysis code.*

**Response:** We recalculated variance inflation factors from the final full-cohort model matrix used in the shared code. The maximum VIF was 1.36. This correction does not alter the interpretation that there was no concerning multicollinearity or any model coefficient.

**Location in the revised manuscript:** Adjusted association, p. 9, lines 153–156.

**Revised text:** ‘The maximum variance inflation factor was 1.36.’

We thank the Editor and Reviewers again for comments that materially improved the transparency, analytical rigor, and interpretation of the manuscript.

Sincerely,

Xiapei Peng，Corresponding Author, on behalf of all authors.
