# Supplementary material for: Association between pre-stroke social participation and current physical resilience across frailty levels among middle-aged and older stroke survivors: A cross-sectional study
Source: PLoS One. 2026 Oct 6;21(10):e0359853. doi: 10.1371/journal.pone.0359853 (PMC13641336; doi:10.1371/journal.pone.0359853)
Supplement: Peer review history

# Peer review: Decision Letter 0

**DOI:** 10.1371/journal.pone.0359853.r001

**Authors:** Marta Ingles

2 Sep 2026

Dear Dr. Peng,

Thank you for submitting your manuscript to PLOS One. After careful consideration, we feel that it has merit but does not fully meet PLOS One’s publication criteria as it currently stands. Therefore, we invite you to submit a revised version of the manuscript that addresses the points raised during the review process.

==============================

oth reviewers found the topic interesting and relevant and recognized several strengths of the study, including the use of validated instruments, the confirmation of stroke diagnosis by neuroimaging, and the potential clinical relevance of examining social participation in relation to post-stroke physical resilience.

However, both reviewers also identified several issues that require substantial clarification and, where feasible, additional analyses. In particular, there are concerns regarding the conceptualization and classification of frailty, the selection and characterization of the study population, the interpretation of the statistical findings, and the limitations inherent to the study design.

==============================

Please submit your revised manuscript by Nov 01 2026 11:59PM. If you will need more time than this to complete your revisions, please reply to this message or contact the journal office at plosone@plos.org. When you're ready to submit your revision, log on to https://www.editorialmanager.com/pone/ and select the 'Submissions Needing Revision' folder to locate your manuscript file.

- A letter that responds to each point raised by the academic editor and reviewer(s). You should upload this letter as a separate file labeled 'Response to Reviewers'.
- A marked-up copy of your manuscript that highlights changes made to the original version. You should upload this as a separate file labeled 'Revised Manuscript with Track Changes'.
- An unmarked version of your revised paper without tracked changes. You should upload this as a separate file labeled 'Manuscript'.

If you would like to make changes to your financial disclosure, please include your updated statement in your cover letter. Guidelines for resubmitting your figure files are available below the reviewer comments at the end of this letter.

If applicable, we recommend that you deposit your laboratory protocols in protocols.io to enhance the reproducibility of your results. Protocols.io assigns your protocol its own identifier (DOI) so that it can be cited independently in the future. For instructions see: https://journals.plos.org/plosone/s/submission-guidelines#loc-laboratory-protocols. Additionally, PLOS One offers an option for publishing peer-reviewed Lab Protocol articles, which describe protocols hosted on protocols.io. Read more information on sharing protocols at https://plos.org/protocols?utm_medium=editorial-email&utm_source=authorletters&utm_campaign=protocols.

As the corresponding author, your ORCID iD is verified in the submission system and will appear in the published article. PLOS supports the use of ORCID, and we encourage all coauthors to register for an ORCID iD and use it as well. Please encourage your coauthors to verify their ORCID iD within the submission system before final acceptance, as unverified ORCID iDs will not appear in the published article. *Only*  the individual author can complete the verification step; PLOS staff *cannot*  verify ORCID iDs on behalf of authors.

We look forward to receiving your revised manuscript.

Kind regards,

Marta Ingles

Academic Editor

PLOS One

Journal Requirements:

When submitting your revision, we need you to address these additional requirements.

1. Please ensure that your manuscript meets PLOS ONE's style requirements, including those for file naming. The PLOS ONE style templates can be found at

https://journals.plos.org/plosone/s/file?id=wjVg/PLOSOne_formatting_sample_main_body.pdf and

https://journals.plos.org/plosone/s/file?id=ba62/PLOSOne_formatting_sample_title_authors_affiliations.pdf

2. Please note that PLOS One has specific guidelines on code sharing for submissions in which author-generated code underpins the findings in the manuscript. In these cases, we expect all author-generated code to be made available without restrictions upon publication of the work. Please review our guidelines at https://journals.plos.org/plosone/s/materials-and-software-sharing#loc-sharing-code and ensure that your code is shared in a way that follows best practice and facilitates reproducibility and reuse.

3. The ethical approval number(s) listed in the manuscript and/or submission metadata does not match the approval number on the ethical approval document you provided. Please ensure that all approval numbers are correct.

4. We notice that your supplementary figures are included in the manuscript file. Please remove them and upload them with the file type 'Supporting Information'. Please ensure that each Supporting Information file has a legend listed in the manuscript after the references list.

5. Please include your full ethics statement in the ‘Methods’ section of your manuscript file. In your statement, please include the full name of the IRB or ethics committee who approved or waived your study, as well as whether or not you obtained informed written or verbal consent. If consent was waived for your study, please include this information in your statement as well

6. We note that you have indicated that there are restrictions to data sharing for this study. PLOS only allows data to be available upon request if there are legal or ethical restrictions on sharing data publicly. For more information on unacceptable data access restrictions, please see http://journals.plos.org/plosone/s/data-availability#loc-unacceptable-data-access-restrictions.

Before we proceed with your manuscript, please address the following prompts:

a) If there are ethical or legal restrictions on sharing a de-identified data set, please explain them in detail (e.g., data contain potentially identifying or sensitive patient information, data are owned by a third-party organization, etc.) and who has imposed them (e.g., a Research Ethics Committee or Institutional Review Board, etc.). Please also provide contact information for a data access committee, ethics committee, or other institutional body to which data requests may be sent.

b) If there are no restrictions, please upload the minimal anonymized data set necessary to replicate your study findings to a stable, public repository and provide us with the relevant URLs, DOIs, or accession numbers. For a list of recommended repositories, please see

https://journals.plos.org/plosone/s/recommended-repositories. You also have the option of uploading the data as Supporting Information files, but we would recommend depositing data directly to a data repository if possible.

We will update your Data Availability statement on your behalf to reflect the information you provide

7. If the reviewer comments include a recommendation to cite specific previously published works, please review and evaluate these publications to determine whether they are relevant and should be cited. There is no requirement to cite these works unless the editor has indicated otherwise.

Additional Editor Comments :

Additional points:

1.Please remove the duplicated statement regarding written informed consent in Section 2.1.

2.Please ensure consistency of the ethics approval information throughout the submission. The submitted materials appear to contain different approval numbers in different sections. The authors should verify the correct approval number and ensure that the same information is reported consistently throughout the manuscript and submission files.

3.Please also ensure consistency in the Data Availability Statement across the submission materials, particularly regarding the ethical restrictions on public sharing of individual-level data and the procedure for reasonable data access requests.

4.Please review the manuscript globally for consistency in terminology, particularly regarding “frail,” “pre-frail/frail,” “physical resilience,” “association,” and “prediction.”

Finally, please consider revising the title if the authors retain the current analytical strategy. The present title refers specifically to “stroke survivors with frailty,” whereas the analytical group includes both pre-frail and frail participants. The title should accurately reflect the population actually studied.

[Note: HTML markup is below. Please do not edit.]

Reviewers' comments:

Reviewer's Responses to Questions

**Comments to the Author**

1. Is the manuscript technically sound, and do the data support the conclusions?

Reviewer #1: Partly

Reviewer #2: Yes

**********

2. Has the statistical analysis been performed appropriately and rigorously?

Reviewer #1: Yes

Reviewer #2: Yes

**********

3. Have the authors made all data underlying the findings in their manuscript fully available?

The PLOS Data policy

Reviewer #1: Yes

Reviewer #2: No

**********

4. Is the manuscript presented in an intelligible fashion and written in standard English?

Reviewer #1: No

Reviewer #2: Yes

**********

Reviewer #1: Thank you for the opportunity to read and review the current manuscript on the association between pre-stroke social participation and post-stroke physical resilience. It is an interesting topic, but I believe the manuscript could be further improved.

Introduction:

The study aims to focus on frail older individuals who have experienced a stroke. Frailty in itself implies low resilience, a higher risk of functional decline, and a poorer prognosis. In your background section, frailty is only mentioned briefly. However, you also state that it is an independent predictor of long-term disability. I suggest that, based on the frailty model you choose to focus on (e.g., Fried? Rockwood?), you need to explain frailty more clearly in the manuscript so that the reader can better understand the significance of the different factors for the outcome.

I believe the problem formulation needs to be strengthened to make the study’s contribution clearer. You need to more clearly justify why participation in social activities prior to stroke may be particularly important for these frail patients in relation to functional ability. Frailty itself is a risk factor for social isolation and reduced participation in society, and it is already well established that frailty is associated with poorer resilience.

In the aim, you refer to an independent association. It is not entirely clear what you mean by this, and how can you determine that the factors you intend to study are independent?

Methods

Clarify your rationale for the chosen inclusion and exclusion criteria. The sample includes individuals aged 45 years and older. However, you frame the study in terms of frailty and also use measures validated for a geriatric population. How do you justify the lower age limit of 45 years in this context?

Furthermore, you have chosen to exclude individuals with a Charlson Comorbidity Index (CCI) score of ≥ 3. Frailty often involves multimorbidity and functional impairment, and a CCI score of 3 or higher is not uncommon in this population. This threshold likely results in the exclusion of the most frail individuals. How did you reason around this decision?

Participants were required to have experienced a stroke at least 3 months prior to inclusion in the study. Additionally, they were asked to assess their social participation 6 months prior to stroke onset. On page 7 (rows 135–137), you state that these timeframes were specifically chosen to minimize recall bias. In what way do these choices achieve that? Please clarify.

I am missing information regarding the frailty assessment. You lack a reference to the validated FRAIL instrument (p. 7, line 140).Which frailty model is the instrument based on, and which domains of the frailty construct does this instrument measure?

You state (p. 8) that you addressed potential covariates, such as chronological age. I would also encourage you to consider frailty and functional ability. Frailty (particularly according to Fried’s phenotype) is strongly associated with impaired physical function, and if the aim is to analyze the impact of social participation on functional ability in frail individuals, this relationship should be considered in the analysis.

Discussion

In the results, a correlation of 0.274 is reported, which should be considered weak according to commonly accepted thresholds for interpreting correlations. I find that a discussion of this result is lacking, please see reference: Schober P, Boer c, schwarte la. correlation coefficients: appropriate use and interpretation. Anesth analg. 2018; 126(5):1763–1768. doi: 10.1213/aNe.0000000000002864.

Furthermore, the discussion needs to be strengthened by providing a balanced and critical interpretation of the findings in relation to frail patients after stroke. This should include consideration of the challenges that frail individuals often already experience regarding social isolation and reduced functional capacity, even prior to stroke onset.

Finally, the clinical implications need to be further developed. What actions can society, healthcare systems, and related sectors take to support older adults’ ability to engage in social participation, thereby enhancing their resilience and improving their capacity to recover from acute illness?

Reviewer #2: This is a cross-sectional study of 314 Chinese stroke patients from a single hospital investigating the association between pre-stroke social participation and post-stroke physical resilience. Frailty, pre-stroke social participation and physical resilience were measured with the FRAIL scale, Social Participation Scale (SPS) and the Physical Resilience Instrument for Older Adults (PRIFOR), respectively. The authors report that 55.4% of the study population were categorized as frail or pre-frail, and higher pre-stroke social participation was independently associated with better physical resilience post-stroke.

The article is well written, and details of ethics approval and informed consent are mentioned. The strengths of the study include confirmation of stroke with neuroimaging and the use of validated scales for measurement of the key variables and outcomes.

The manuscript could be strengthened by addressing several limitations.

1. This is a single centre, hospital-based study, and the data cannot be generalized to community-dwelling stroke survivors and to other populations.

2. The cross-sectional design and retrospective assessment of pre-stroke resilience have their inherent limitations. These are briefly mentioned by the authors but would need discussion in greater detail.

3. At what time point post-stroke were frailty and resilience assessed? This may have important implications to the degree of frailty and resilience.

4. Both pre-frail and frail patients (as per the FRAIL scale) were lumped together in the study. They are referred to as the ‘frail group’ in the manuscript – this is incorrect, and they should be referred to as the ‘pre-frail/ frail group’.

5. Can the authors justify analyzing the pre-frail and frail patients together? It is likely that resilience would be lower in the frail group compared to the non-frail group.

6. Physical resilience is the opposite of frailty. Including only pre-frail/frail patients would automatically lead to a finding of lower levels of resilience. The authors need to study participants across the frailty spectrum (non-frail, pre-frail and frail) to meaningfully describe the association between pre-stroke social participation and post-stroke physical resilience. Was the association between pre-stroke social participation and post-stroke physical resilience similar in non-frail survivors? Suggest stratifying analysis by frailty severity (non-frail vs pre-frail vs frail).

7. Pre-stroke level of physical activity/ functioning is a confounding factor that is likely to have a major impact on post-stroke resilience. This was not assessed, and should be discussed.

8. Other confounders such as treatment received, timing of treatment, intensity and duration of rehabilitation and availability of community support services, which may impact post-stroke recovery and resilience, were not assessed.

9. Minor comment: Under methods, 2.1 - sentence regarding informed consent is duplicated – lines 98-99 and 100-101.

**********

what does this mean?). If published, this will include your full peer review and any attached files.

If you choose “no”, your identity will remain anonymous but your review may still be made public.

**Do you want your identity to be public for this peer review?**  For information about this choice, including consent withdrawal, please see our Privacy Policy

Reviewer #1: No

Reviewer #2: No

**********

[NOTE: If reviewer comments were submitted as an attachment file, they will be attached to this email and accessible via the submission site. Please log into your account, locate the manuscript record, and check for the action link "View Attachments". If this link does not appear, there are no attachment files.]

To ensure your figures meet our technical requirements, please review our figure guidelines: https://journals.plos.org/plosone/s/figures

You may also use PLOS’s free figure tool, NAAS, to help you prepare publication quality figures: https://journals.plos.org/plosone/s/figures#loc-tools-for-figure-preparation.

NAAS will assess whether your figures meet our technical requirements by comparing each figure against our figure specifications.

# Author comment: Author response to Decision Letter 1

**DOI:** 10.1371/journal.pone.0359853.r002

7 Sep 2026

We sincerely thank the Editor and Reviewers for their constructive comments. We have revised the manuscript thoroughly and addressed every comment point by point. The principal revisions include clarification of the study design and ethics information; analysis of all 314 participants across the non-frail, pre-frail, and frail categories; additional interaction and sensitivity analyses; clarification of time since stroke and the seven social participation items; revision of the interpretation of correlation magnitude; expansion of the limitations and clinical implications; correction and renumbering of the references; and preparation of the analysis code and Supporting Information files. A detailed point-by-point Response to the Editor and Reviewers, including the locations and revised text, is uploaded as a separate file.

Attachment Submitted filename: Response to Reviewers.docx

# Peer review: Decision Letter 1

**DOI:** 10.1371/journal.pone.0359853.r003

**Authors:** Marta Ingles

21 Sep 2026

Association between pre-stroke social participation and current physical resilience across frailty levels among middle-aged and older stroke survivors: A cross-sectional study

PONE-D-26-23300R1

Dear Dr. Peng

We’re pleased to inform you that your manuscript has been judged scientifically suitable for publication and will be formally accepted for publication once it meets all outstanding technical requirements.

Within one week, you’ll receive an e-mail detailing the required amendments. When these have been addressed, you’ll receive a formal acceptance letter and your manuscript will be scheduled for publication.

An invoice will be generated when your article is formally accepted. Please note, if your institution has a publishing partnership with PLOS and your article meets the relevant criteria, all or part of your publication costs will be covered. Please make sure your user information is up-to-date by logging into Editorial Manager at Editorial Manager® and clicking the ‘Update My Information' link at the top of the page. For questions related to billing, please contact billing support.

If your institution or institutions have a press office, please notify them about your upcoming paper to help maximize its impact. If they’ll be preparing press materials, please inform our press team as soon as possible -- no later than 48 hours after receiving the formal acceptance. Your manuscript will remain under strict press embargo until 2 pm Eastern Time on the date of publication. For more information, please contact onepress@plos.org.

Kind regards,

Marta Ingles

Academic Editor

PLOS One

Additional Editor Comments (optional):

Reviewers' comments:

Reviewer's Responses to Questions

**Comments to the Author**

Reviewer #1: All comments have been addressed

Reviewer #2: All comments have been addressed

**********

2. Is the manuscript technically sound, and do the data support the conclusions?

Reviewer #1: Yes

Reviewer #2: Yes

**********

3. Has the statistical analysis been performed appropriately and rigorously?

Reviewer #1: Yes

Reviewer #2: Yes

**********

4. Have the authors made all data underlying the findings in their manuscript fully available?

The PLOS Data policy

Reviewer #1: Yes

Reviewer #2: Yes

**********

5. Is the manuscript presented in an intelligible fashion and written in standard English?

Reviewer #1: Yes

Reviewer #2: Yes

**********

Reviewer #1: Thank you for this revised manuscript. The authors have made substantial improvements, and my concerns have been adequately addressed.

For your information, I noticed that the instrument PRIFOR is not written out in full the first time it is mentioned (cf. lines 82 and 89).

Thank you for an enjoyable and interesting read. I wish the authors all the best with their work.

Reviewer #2: (No Response)

**********

what does this mean?). If published, this will include your full peer review and any attached files.

If you choose “no”, your identity will remain anonymous but your review may still be made public.

**Do you want your identity to be public for this peer review?**  For information about this choice, including consent withdrawal, please see our Privacy Policy

Reviewer #1: No

Reviewer #2: No

**********

# Editor report: Acceptance letter

**DOI:** 10.1371/journal.pone.0359853.r004

**Authors:** Marta Ingles

PONE-D-26-23300R1

PLOS One

Dear Dr. Peng,

I'm pleased to inform you that your manuscript has been deemed suitable for publication in PLOS One. Congratulations! Your manuscript is now being handed over to our production team.

At this stage, our production department will prepare your paper for publication. This includes ensuring the following:

* All references, tables, and figures are properly cited

* All relevant supporting information is included in the manuscript submission,

* There are no issues that prevent the paper from being properly typeset

You will receive further instructions from the production team, including instructions on how to review your proof when it is ready. Please keep in mind that we are working through a large volume of accepted articles, so please give us a few days to review your paper and let you know the next and final steps.

Lastly, if your institution or institutions have a press office, please let them know about your upcoming paper now to help maximize its impact. If they'll be preparing press materials, please inform our press team within the next 48 hours. Your manuscript will remain under strict press embargo until 2 pm Eastern Time on the date of publication. For more information, please contact onepress@plos.org.

You will receive an invoice from PLOS for your publication fee after your manuscript has reached the completed accept phase. If you receive an email requesting payment before acceptance or for any other service, this may be a phishing scheme. Learn how to identify phishing emails and protect your accounts at https://explore.plos.org/phishing.

If we can help with anything else, please email us at customercare@plos.org.

Thank you for submitting your work to PLOS One and supporting open access.

Kind regards,

PLOS One Editorial Office Staff

on behalf of

Dr. Marta Ingles

Academic Editor

PLOS One
